# Montmorillonite-Reinforced Acrylic Copolymer Adhesive for Robust Underwater Bonding via Bulk-Interfacial Adhesion Synergy

**DOI:** 10.3390/polym18172129

**Published:** 2026-08-31

**Authors:** Wenhui Li, Xiaoxuan Xue, Zhan Gao, Yizhang Yang, Bairan Chen, Chao Yang

**Affiliations:** College of Materials Science and Engineering, Shandong Key Laboratory of Low Dimensional Materials and Polymer Composites, Qingdao University, Qingdao 266071, China

**Keywords:** underwater adhesion, acrylic copolymer, montmorillonite, solvent exchange

## Abstract

Underwater adhesion is often compromised by interfacial hydration and insufficient bulk properties. Herein, a montmorillonite-reinforced poly (acrylic acid-co-butyl acrylate-co-2-phenoxyethyl acrylate) [P(AA-co-BA-co-PEA)/MMT] liquid adhesive was fabricated by one-pot free-radical polymerization. Solvent exchange and the balanced hydrophilic–hydrophobic composition facilitated hydration-layer displacement and intimate substrate contact, whereas MMT introduced additional physical interactions that restricted chain mobility and reinforced the adhesive bulk. The adhesive achieved underwater lap-shear strengths of 66.01–238.00 kPa on polypropylene, poly (vinyl chloride), polytetrafluoroethylene, wood, 304 stainless steel, and glass, representing improvements of 10.35–157.62% over the MMT-free adhesive. The highest strength, 238.00 ± 5.52 kPa, was obtained on 304 stainless steel. Moreover, the 180° peel strength increased by 23.90% to 107.76 ± 5.12 N m^−1^, while the swelling ratio decreased by 33.59% to 10.20 ± 1.09%. Rheological and thermal analyses further supported the MMT-induced enhancement of the adhesive bulk. This synergistic regulation of interfacial adhesion and composite reinforcement provides a simple route toward versatile liquid adhesives for robust underwater bonding.

## 1. Introduction

Adhesives are materials capable of bonding two objects together, transferring the load applied to one substrate to the other, thereby preventing separation and enabling either reversible or permanent bonding [1,2,3]. Many conventional adhesives exhibit satisfactory adhesion strength only on dry solid surfaces in air. However, under humid conditions, the hydration layer present at the substrate interface macroscopically reduces the contact area between the adhesive and the substrate, severely hindering interfacial bonding and diminishing adhesive strength. Moreover, prolonged exposure to water may cause swelling, degradation, plasticization, hydrolysis, and even decomposition of the adhesive, leading to bond failure [4,5]. Nevertheless, in numerous practical scenarios, the challenge of humid environments is unavoidable. For instance, biomedical adhesives inevitably come into contact with sweat or blood during use, while applications such as underwater sensors, subaqueous pipeline repair, and marine engineering often involve long-term exposure or even complete immersion in water. Consequently, the development of adhesives that maintain excellent performance under humid or fully submerged conditions has become an important and promising direction in the field of adhesive research [6,7,8].

Achieving strong adhesion in aqueous environments poses considerably more challenges than in air. When most materials are exposed to water, a hydration layer forms on their surfaces, which reduces the contact area between the adhesive and the substrate and impedes their interfacial interactions [9,10]. Furthermore, water penetration into the adhesive can induce swelling and aging, ultimately leading to loss of adhesion. Therefore, to realize underwater adhesion, it is first necessary to disrupt the hydration layer [11].

Therefore, how to remove the hydration layer has become the primary challenge for underwater adhesion. Two main strategies are generally adopted: repelling the hydration layer and absorbing the hydration layer, with the former being more commonly employed [12,13]. Absorbing the interfacial water to dry the substrate surface and promote contact between the adhesive and the substrate also represents a feasible approach [14]. Solvent exchange has attracted considerable attention as a strategy to disrupt the hydration layer, providing inspiration for the preparation of underwater adhesives [15,16,17,18]. In this strategy, a water-miscible organic good solvent is chosen as the adhesive solvent. Upon contact with water, the solvent diffuses into the aqueous phase, creating a concentration-gradient-driven solvent exchange process. During this diffusion, the hydration layer at the adhesive–substrate interface is displaced, forming a transiently dried interfacial layer that minimizes water interference with adhesion. Subsequently, the adhesive spreads over the substrate surface, establishing sufficient contact and forming adhesion through intermolecular interactions. As the good solvent further diffuses, the adhesive concentration increases, inducing polymer precipitation, hydrophobic aggregation [18], or physical crosslinking, thereby achieving solidification. For instance, Ye et al. employed a DMSO–water solvent exchange strategy to develop a novel glucose-based copolymer underwater gelling material, which exhibited excellent shear adhesion strength on various surfaces including plastics, metals, glass, and pigskin [19].

Once the hydration layer is displaced, the interfacial adhesion between the adhesive and the substrate becomes a critical factor influencing the overall adhesive strength. Interfacial adhesion can arise from various mechanisms, which are generally categorized into mechanical interlocking [20,21,22], covalent bonding [23,24], and non-covalent interactions.

Mechanical interlocking is effective only on substrates with rough surfaces, where the adhesive penetrates into the surface irregularities, and the adhesive strength is often proportional to the surface roughness. Covalent bonding represents the most common and strongest form of bonding, providing high bond energy and thus significantly enhancing the interfacial adhesion and overall strength [25]. However, for substrates such as metals, plastics, and glass, which lack surface functional groups capable of covalent bonding with the adhesive and also lack voids for mechanical interlocking, alternative interactions are required. These include non-covalent interactions such as metal coordination [26,27], hydrophobic association [8,28], π–π stacking [29], host–guest interactions [30,31], electrostatic interactions [32], hydrogen bonding [33,34,35], dipole–dipole interactions [36], cation–π interactions [37,38,39,40], or synergistic supramolecular interactions involving multiple non-covalent bonds [8,41].

The adhesion performance of an adhesive is governed by both its bulk properties and its interfacial adhesion behavior. The bulk properties of the adhesive, namely its resistance to deformation and failure, depend on the physically crosslinked network arising from polymer-chain entanglements. The synergistic matching of these two aspects is essential for achieving optimal adhesion performance [42]. Montmorillonite (MMT), a layered silicate mineral with a large specific surface area, good dispersibility, and high adsorption capacity, has been widely used for polymer reinforcement. Intercalation of polymer chains into the MMT galleries forms nanoscale composites, significantly enhancing the bulk mechanical properties of the polymer [43]. For instance, Ji et al. [44] prepared a hydrogel with anti-swelling properties and underwater self-adhesion by modifying MMT with tannic acid intercalation, in which MMT served as physical crosslinking nodes to significantly reinforce the network structural integrity of the hydrogel, enabling it to maintain stable adhesion in underwater environments.

Polyacrylate-based materials have been widely used as underwater adhesives due to their excellent adhesion strength and water resistance [45], motivating extensive modifications of acrylic adhesive systems. Acrylic monomers contain both hydrophobic and hydrophilic groups, enabling the design of adhesives that exploit hydrophilic–hydrophobic synergy to disrupt the hydration layer and achieve robust adhesion. Yang et al. [8] demonstrated this strategy by developing a supramolecular adhesive composed of hydrophobic copolymers (hexyl acrylate and styrene) and hydrophilic acrylic acid, achieving underwater adhesion strengths of 92–116 kPa on various substrates. Building upon this concept, dissolving amphiphilic copolymers in good organic solvents enables solvent exchange with water in underwater environments, providing a versatile synthetic route for high-performance underwater adhesives [33]. Liu et al. [46] prepared non-swelling, high-strength underwater adhesives via random copolymerization of acrylic acid, butyl acrylate, and adenine acrylate in DMSO. Through DMSO–water solvent exchange-induced hydrophobic aggregation, they achieved adhesion strengths of 34–72 kPa on PTFE, plastics, metals, rubber, and glass.

However, the above strategies have mainly focused on optimizing interfacial adhesion, with insufficient attention given to the synergistic balance between bulk properties and interfacial adhesion. Moreover, studies combining solvent exchange with MMT-reinforced acrylic systems remain relatively limited, and the resulting underwater adhesion strengths are still comparatively low. To address this, the present work, based on acrylic systems and in combination with the solvent exchange strategy, employs a terpolymerization of acrylic acid (AA), butyl acrylate (BA), and 2-phenoxyethyl acrylate (PEA) via one-pot free-radical polymerization to prepare a P(AA-co-BA-co-PEA) underwater adhesive. In this design, AA provides hydrophilic carboxyl groups for metal coordination and surface wetting; BA contributes hydrophobic long alkyl chains for hydration-layer displacement and chain flexibility; and PEA introduces benzene rings that enable π–π stacking interactions to improve the bulk properties of the adhesive. To further enhance the adhesive, sodium montmorillonite (MMT) was introduced as a nanomaterial into the poly(acrylic acid)-based ternary system, serving as physical crosslinking nodes to structurally increase chain entanglement, thereby yielding the MMT-modified P(AA-co-BA-co-PEA)/MMT underwater adhesive. The chemical compositions were determined by FTIR and NMR; the molecular weight was characterized by gel permeation chromatography (GPC); the interaction between MMT and polymer chains was characterized by XRD. Underwater adhesion strengths on various substrates were evaluated by lap-shear tests, examining the adhesion and peel strengths on six different substrates, namely PP, PVC, PTFE, wood, 304 stainless steel, and glass slides, and the swelling ratios were compared. Thermal and rheological properties were characterized by simultaneous thermal analysis and rotational rheometry, respectively. On this basis, the mechanism by which the incorporation of nanomaterials improves adhesion performance was discussed.

## 2. Materials and Methods

### 2.1. Materials

Acrylic acid (AA, GC), butyl acrylate (BA, AR), 2-phenoxyethyl acrylate (PEA, 90%), N,N-dimethylformamide (DMF, AR), azobisisobutyronitrile (AIBN, 98%), and sodium montmorillonite (MMT) were purchased from Shanghai Macklin Biochemical Co., Ltd. (Shanghai, China). Deionized water was obtained from Qingdao Chengda Distilled Water Co., Ltd. (Qingdao, China). Polypropylene (PP), polyvinyl chloride (PVC), polytetrafluoroethylene (PTFE), basswood, 304 stainless steel, glass slides, and polyethylene terephthalate (PET) film were purchased from Qingdao Hongfengxin Environmental Engineering Co., Ltd. (Qingdao, China).

### 2.2. Preparation of P(AA-co-BA-co-PEA)

Accurately weighed 2.162 g of AA (30 mmol), 4.486 g of BA (35 mmol), and 6.727 g of PEA (35 mmol) were mixed and dissolved in 25 mL of DMF. In a separate vessel, 0.2675 g of AIBN (1.629 mmol, 2 wt% of total monomers) was dissolved in 50 mL of DMF. This initiator solution was combined with the monomer solution in a 500 mL round-bottom flask, and the mixture was stirred at 60 °C for 16 h. After the reaction, the resulting solution was concentrated to 15 mL by rotary evaporation at 75–80 °C under reduced pressure (~1 kPa).

### 2.3. Preparation of P(AA-co-BA-co-PEA)/MMT

A two-step method was employed to prepare the MMT dispersions. Sodium montmorillonite (MMT) was first dried at 60 °C under vacuum for 5 h to remove moisture. The dried MMT powder was then added to DMF, and the mixture was magnetically stirred for 30 min to obtain a pre-dispersion, followed by ultrasonication in an ultrasonic cleaner for 2 h. After sonication, the dispersions were allowed to stand for 2 h, and the upper homogeneous fractions were collected. The MMT loading was varied (1, 3, and 5 g) while maintaining a constant DMF volume of 50 mL, and each dispersion was prepared using the same procedure. All three MMT dispersions (1, 3, and 5 g) remained homogeneous after standing. The 5 g MMT formulation was selected for subsequent experiments based on its superior adhesion strength under identical testing conditions (Figure S1).

Subsequently, all preparation procedures for P(AA-co-BA-co-PEA)/MMT were identical to those described above for P(AA-co-BA-co-PEA), except that pure DMF was replaced with a DMF dispersion containing MMT.

### 2.4. Characterization

The chemical structures and compositions of pristine MMT, P(AA-co-BA-co-PEA), and P(AA-co-BA-co-PEA)/MMT were characterized using a Fourier transform infrared spectrometer (Thermo Scientific iS5, Thermo Fisher Scientific Inc., Waltham, MA, USA) equipped with an attenuated total reflection (ATR) accessory. Spectra were recorded over a wavenumber range of 400–4000 cm^−1^ with a resolution of 4 cm^−1^ and 32 scans.

The copolymer composition was determined using a nuclear magnetic resonance spectrometer (Bruker Avance 600, Bruker Corporation, Billerica, MA, USA) with deuterated chloroform (CDCl_3_) as the solvent.

The molecular weight of the P(AA-co-BA-co-PEA) adhesive was determined using a gel permeation chromatograph (Agilent 1260, Agilent Technologies Inc., Santa Clara, CA, USA). DMF was used as the mobile phase, and the injection volume was 20 μL. Narrow-distribution poly(methyl methacrylate) (PMMA) standards were used for calibration. The calibration curve was established by plotting the logarithm of molecular weight (log *M*) against the elution volume (*V_e_*) and fitted using a first-order polynomial least-squares regression: log *M* = −0.809476*V_e_* + 10.8073 (*R*^2^ = 0.9969). The molecular weight and dispersity were calculated using the GPC software.

X-ray diffraction (XRD) patterns were recorded using a (D2-PHASER, Bruker AXS GmbH, Karlsruhe, Germany) diffractometer with Cu Kα radiation (λ = 0.154 nm) at 40 kV and 40 mA. The diffraction patterns of pristine MMT, P(AA-co-BA-co-PEA), and P(AA-co-BA-co-PEA)/MMT were collected over a 2θ range of 3° to 10° at a scanning speed of 1°/min.

### 2.5. Thermal Analysis

The thermal properties of the P(AA-co-BA-co-PEA) and P(AA-co-BA-co-PEA)/MMT adhesives before and after solidification in water for 5 min were characterized using a simultaneous thermal analyzer (NETZSCH STA 449F3, Netzsch-Gerätebau GmbH, Selb, Germany) under a nitrogen atmosphere. The samples were heated from 25 to 800 °C at a heating rate of 10 °C/min. DSC, TG, and DTG curves were obtained simultaneously to evaluate the thermal performance.

### 2.6. Rheological Measurement

Rheological properties of P(AA-co-BA-co-PEA) and P(AA-co-BA-co-PEA)/MMT adhesives before and after solidification in water for 5 min were analyzed using a rotational rheometer in oscillatory mode with frequency sweep. The tests were conducted at 25 °C with an angular frequency range of 0.1–100 rad/s. Storage modulus (G′), loss modulus (G″), and complex viscosity (η) were recorded.

### 2.7. Adhesion Performance

**Figure 1:**
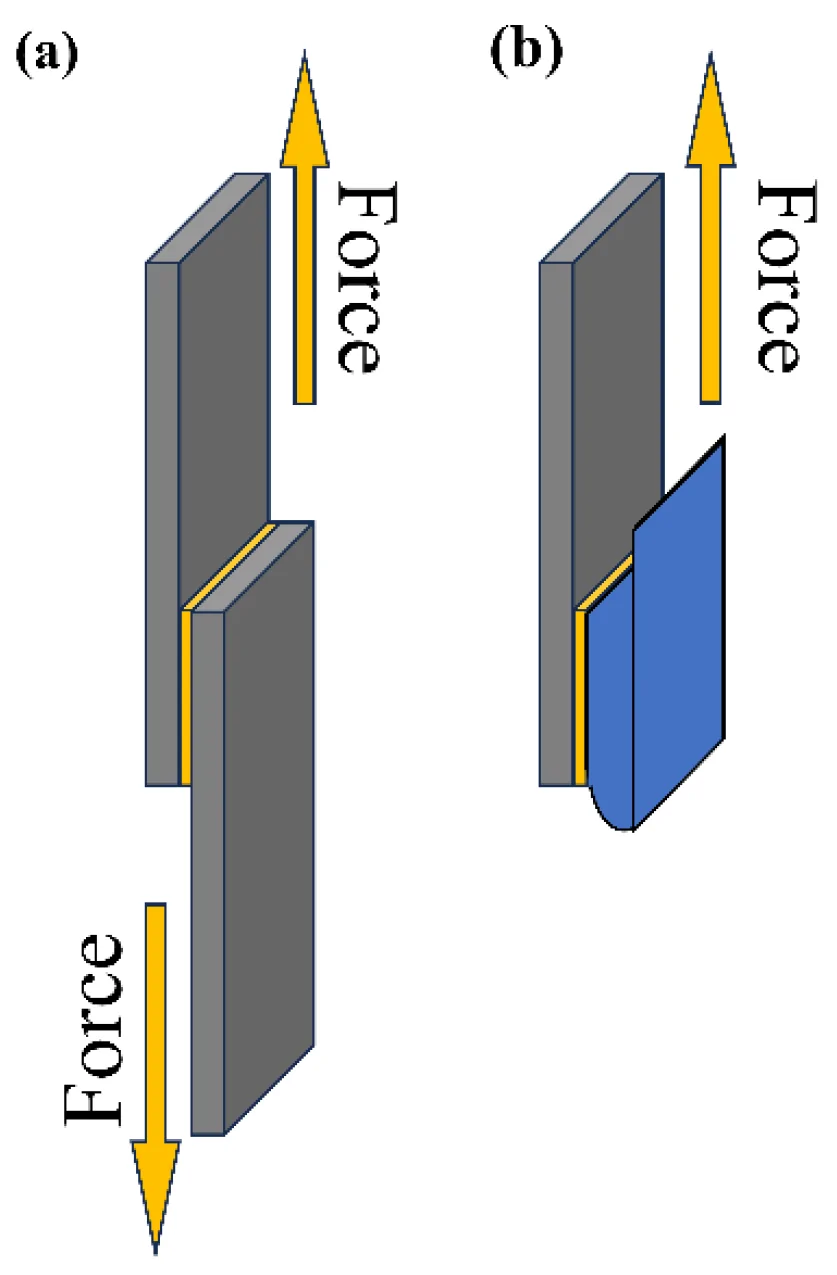
Schematic illustration of adhesion performance tests: (**a**) lap-shear test; (**b**) peel test.

The adhesive strength on various substrates, the change in adhesion strength upon repeated use, and the peel strength were evaluated using a universal testing machine (Instron 5300, Instron Corporation, Norwood, MA, USA), as illustrated in Figure 1.

Lap shear tests were employed to measure the adhesive strength. Briefly, two identical substrate strips were presoaked in water. One substrate was taken out, and 0.1 mL of the adhesive was uniformly applied onto a 25 × 25 mm^2^ area near the bottom of its surface. The top of the second wet substrate was then placed onto the coated area, forming a bonded area of 25 × 25 mm^2^. The assembly was immersed in water, and a 500 g weight was placed on the bonded area to temporarily fix the substrates. After 10 min, the weight was removed, and the assembly was further immersed for 12 h. Adhesive strength was then measured using the Instron 5300 universal testing machine at a tensile speed of 10 mm/min. The maximum force during pulling was recorded to calculate the underwater adhesion strength (Figure 1a). Substrates used in the lap shear tests included PP, PVC, PTFE, basswood, 304 stainless steel, and glass slides, with dimensions of 25 × 75 × 1.5 mm^3^. The failure mode of each fracture was determined by visual inspection.

After the initial lap shear test, the two substrates were completely separated. They were then re-bonded under water with the same bonded area of 25 × 25 mm^2^. A 500 g weight was placed on the bonded area to apply pressure. After 5 min of immersion, the adhesive strength was measured again. This process was repeated five times to evaluate the change in adhesion strength upon repeated use on the six different substrates.

For peel strength testing, a PTFE plate with very low surface energy was used as the substrate. After applying the adhesive onto a 25 × 25 mm^2^ area on the wet PTFE plate, a PET film (25 × 75 mm^2^) was placed over the adhesive. The solidification procedure was the same as that in the lap shear test. Peel strength was then measured using the Instron 5300 universal testing machine at a peel angle of 180° and a tensile speed of 10 mm/min, as shown in Figure 1b.

### 2.8. Swelling Ratio

A certain amount of the adhesive was weighed and recorded as *W*_1_. The sample was then immersed in deionized water, and the water was replaced every hour. After 12 h, the adhesive was taken out, wiped dry to remove surface water, and weighed again, recorded as *W*_2_. The swelling ratio was calculated according to Equation (1):

(1) $$SR=\frac{W_{2}-W_{1}}{W_{1}}\times 100\%$$

### 2.9. Statistical Analysis

All experiments were performed at least three times to obtain the mean values, and the standard deviations were calculated. All data are presented as mean ± standard deviation (SD).

## 3. Results and Discussion

### 3.1. Synthesis and Characterization of the Adhesives

**Figure 2:**
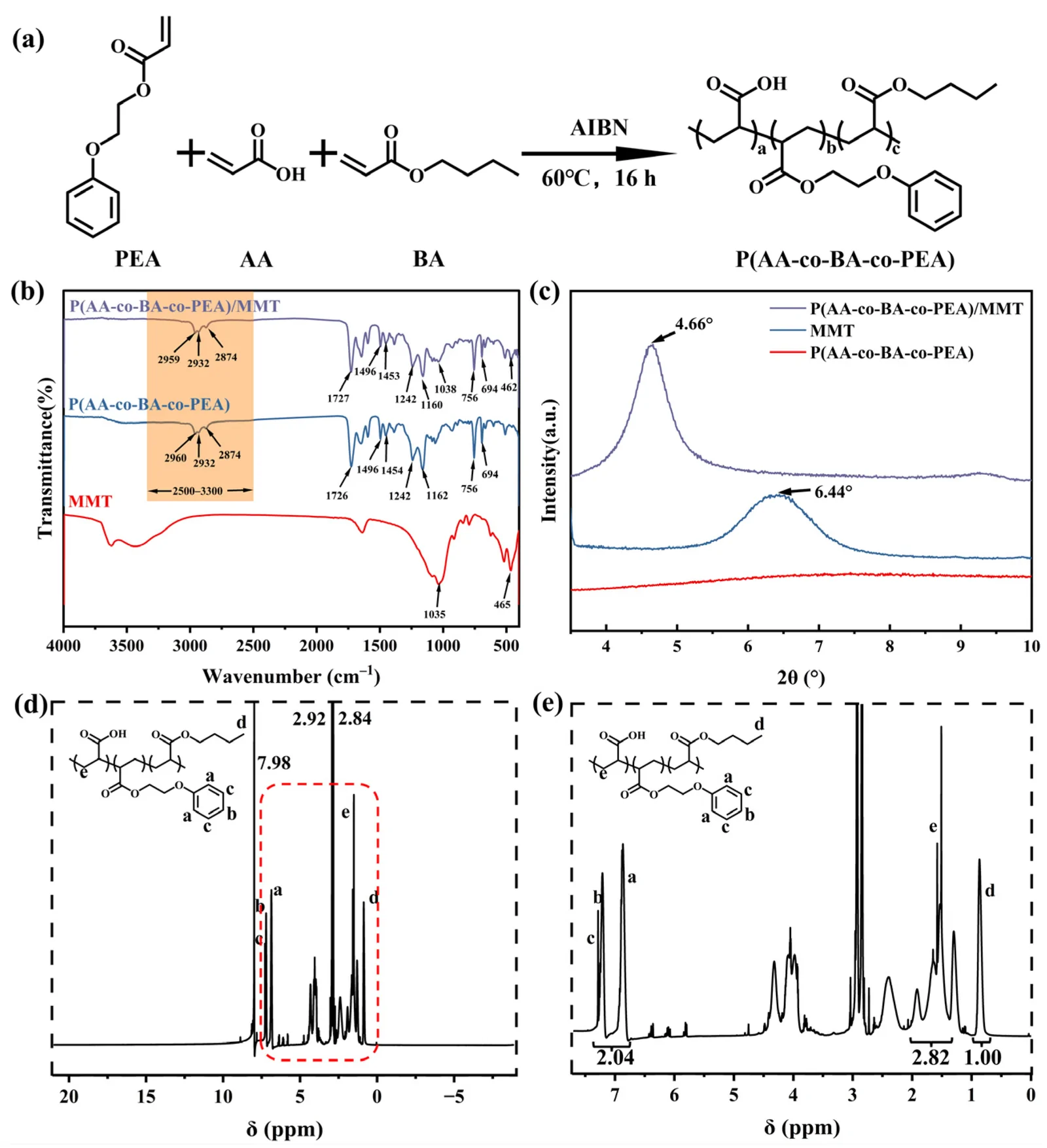
Synthesis and characterization of the adhesives: (**a**) Schematic illustration of the synthesis of the adhesives; FTIR spectra (**b**) and XRD patterns (**c**) of MMT, P(AA-co-BA-co-PEA), and P(AA-co-BA-co-PEA)/MMT, respectively; (**d**) ^1^H NMR spectrum of P(AA-co-BA-co-PEA); (**e**) enlarged ^1^H NMR spectrum of P(AA-co-BA-co-PEA) with integration values. The lowercase letters in (**d**,**e**) indicate the proton signals corresponding to the respective chemical shifts; detailed peak assignments are provided in the main text (Section 3.1).

The P(AA-co-BA-co-PEA) and P(AA-co-BA-co-PEA)/MMT adhesives were prepared via a one-pot method (Figure 2a) and characterized by Fourier transform infrared (FTIR) spectroscopy, with the results shown in Figure 2b. In the spectrum of P(AA-co-BA-co-PEA), characteristic absorption peaks corresponding to the functional groups of the AA, BA, and PEA units were clearly observed. Specifically, the peaks at 2874 cm^−1^, 2932 cm^−1^, and 2960 cm^−1^ are attributed to the symmetric stretching vibration of methylene (–CH_2_–), and the symmetric and asymmetric stretching vibrations of methyl (–CH_3_), respectively, which are characteristic of the BA units. The peaks at 756 cm^−1^ and 694 cm^−1^ correspond to the out-of-plane C–H bending vibrations of the monosubstituted benzene ring in the PEA units. The peaks at 1454 cm^−1^ and 1496 cm^−1^ are assigned to the symmetric and asymmetric vibrations of the C=C skeleton in the phenyl ring of PEA, respectively. The peaks at 1726 cm^−1^, 1242 cm^−1^, and 1162 cm^−1^ are attributed to the C=O stretching vibration of the ester group, and the asymmetric and symmetric stretching vibrations of the C–O bond in the ester group, respectively. In addition, a broad peak in the range of 2500–3300 cm^−1^ is observed, corresponding to the O–H stretching vibration of the carboxyl group from the AA units.

Compared with the spectrum of P(AA-co-BA-co-PEA), the FTIR spectrum of P(AA-co-BA-co-PEA)/MMT exhibited similar characteristic absorption peaks, such as those at 1160 cm^−1^ and 1242 cm^−1^ (asymmetric and symmetric C–O stretching of the ester group) and at 1727 cm^−1^ (C=O stretching of the ester group). The peaks at 2874 cm^−1^, 2932 cm^−1^, and 2959 cm^−1^ correspond to the symmetric stretching vibration of methylene (–CH_2_–), and the symmetric and asymmetric stretching vibrations of methyl (–CH_3_) from the BA units, while the four characteristic peaks of the phenyl ring in the PEA units were observed at 756 cm^−1^, 694 cm^−1^, 1453 cm^−1^, and 1496 cm^−1^. Notably, the broad band in the region of 2500–3300 cm^−1^ showed a noticeable decrease in intensity, which is attributed to the interaction between the carboxyl groups of the copolymer and the MMT nanolayers. In addition, two characteristic peaks of MMT were observed at 1038 cm^−1^ and 465 cm^−1^, corresponding to the asymmetric stretching and bending vibrations of Si–O–Si, respectively. These peaks, combined with the FTIR spectrum of pristine MMT, confirm the successful incorporation of MMT into the polymer matrix.

Notably, in the FTIR spectra of both adhesives, no obvious absorption peak was observed in the region of 1600–1640 cm^−1^, which is characteristic of the terminal vinyl C=C stretching vibration of acrylate monomers (including AA, BA, and PEA). This observation indicates that the three monomers were substantially consumed during the free-radical copolymerization and subsequent rotary evaporation, confirming the successful preparation of the P(AA-co-BA-co-PEA) copolymer backbone.

To confirm the intercalation structure of MMT in the polymer matrix, XRD analysis was performed on pristine MMT, P(AA-co-BA-co-PEA), and P(AA-co-BA-co-PEA)/MMT, with the results shown in Figure 2c. Pristine MMT exhibited a distinct diffraction peak at 2θ = 6.44°, corresponding to a basal spacing of approximately 1.37 nm. After the incorporation of MMT into the P(AA-co-BA-co-PEA) matrix, this characteristic diffraction peak shifted significantly to a lower angle of 2θ = 4.66°, with the corresponding interlayer spacing increasing to approximately 1.89 nm. The substantial expansion of the MMT interlayer spacing indicates that polymer chains have successfully intercalated into the galleries between MMT layers, leading to the expansion of the layered structure and confirming the formation of an intercalated structure in the composite. Meanwhile, the pristine P(AA-co-BA-co-PEA) exhibited no obvious diffraction peaks in this low-angle region, further indicating that the diffraction peak at 4.66° in the composite primarily originates from the structural changes of the MMT interlayers. These results demonstrate that the polymer chains have been successfully intercalated into the interlayer galleries of MMT, forming a nanocomposite structure.

The P(AA-co-BA-co-PEA) adhesive was characterized by ^1^H NMR spectroscopy, and the results are shown in Figure 2d. In the ^1^H NMR spectrum, the symmetric doublets at 2.84 ppm and 2.92 ppm are assigned to the methyl protons of the DMF solvent, and the peak at 7.98 ppm corresponds to the aldehyde proton of DMF. The copolymer composition was determined by ^1^H NMR integration. The methyl protons of BA (0.86 ppm, 3H) and the aromatic protons of PEA (6.86–7.28 ppm, 5H) were used as characteristic signals, with their integrals set as 1.00 and 2.04, respectively. Thus, the molar ratios of BA and PEA units were calculated as BA = 1.00/3 = 0.333 and PEA = 2.04/5 = 0.408. The AA content was obtained by difference: AA = 1 − 0.333 − 0.408 = 0.259. Accordingly, the molar composition of the copolymer was determined as AA:BA:PEA = 25.9:33.3:40.8. The integral of the backbone methylene region (1.4–2.1 ppm, 2.82) was consistent with the expected contribution from total main-chain protons, serving as an internal consistency check. Compared with the feed molar ratio (30:35:35), the PEA content in the copolymer was significantly higher, while the AA content was lower.

The molecular weight of the P(AA-co-BA-co-PEA) adhesive was determined by gel permeation chromatography (GPC). The average molecular weight (M_n_) number was found to be 10,617 Da with a polydispersity (PDI = Mw/Mn) of 2.9, indicating the occurrence of free radical polymerization. Combined with the characteristic functional group peaks observed in the FTIR spectra and the corresponding proton signals in the ^1^H NMR spectra, these results collectively confirm the successful preparation of both P(AA-co-BA-co-PEA) and P(AA-co-BA-co-PEA)/MMT via one-pot free radical copolymerization.

### 3.2. Adhesion Performance on Various Substrates

To comprehensively evaluate the underwater adhesion performance of the adhesives, lap shear tests on various substrates and 180° peel tests were conducted to assess the adhesion strength, reusability, and peel resistance, and to elucidate the effect of MMT incorporation on the overall adhesion performance.

**Figure 3:**
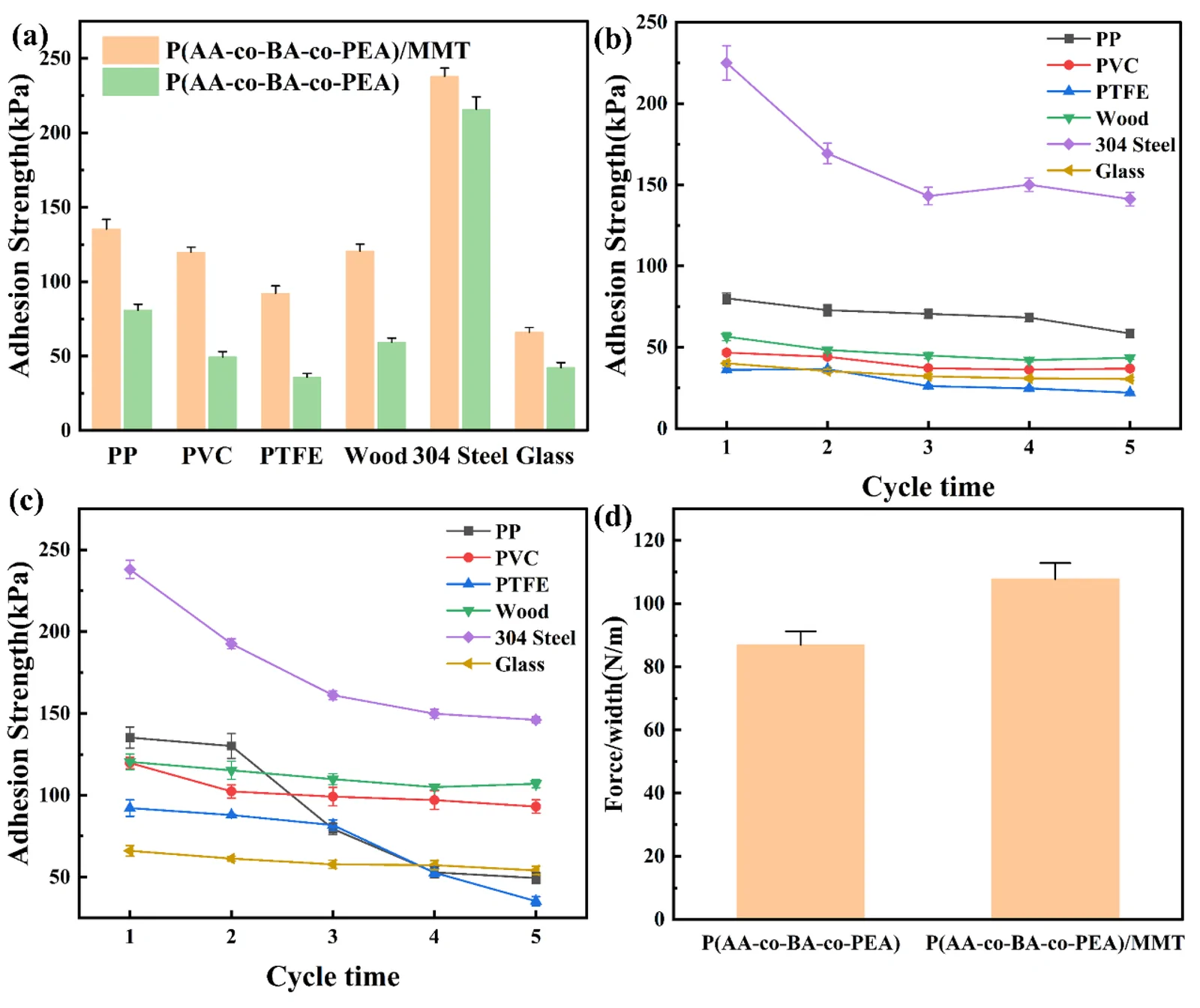
Underwater adhesion strengths: (**a**) adhesion strength of P(AA-co-BA-co-PEA) and P(AA-co-BA-co-PEA)/MMT on various substrates; (**b**) reusability of P(AA-co-BA-co-PEA) adhesive; (**c**) reusability of P(AA-co-BA-co-PEA)/MMT adhesive; (**d**) peel strength of P(AA-co-BA-co-PEA) and P(AA-co-BA-co-PEA)/MMT.

In the lap-shear tests of P(AA-co-BA-co-PEA), visual observation of the two fracture surfaces revealed that both were covered with adhesive residue, and thus the failure mode was identified as cohesive failure. The results showed that P(AA-co-BA-co-PEA) exhibited certain adhesion strength on all tested substrates (Figure 3a), with values of 80.78 ± 3.96 kPa, 49.45 ± 3.55 kPa, 35.77 ± 2.65 kPa, 59.32 ± 2.84 kPa, 215.68 ± 8.44 kPa, and 42.24 ± 3.38 kPa on PP, PVC, PTFE, wood, 304 stainless steel, and glass slides, respectively. This is attributed to the carboxyl groups of AA, which serve as hydrophilic groups providing a certain degree of hydrophilicity. In synergy with the hydrophobic monomers, they can more effectively displace the interfacial hydration layer. As polar groups, they also enhance adhesion to metals and plastics [47]. Meanwhile, the benzene rings of PEA and the long alkyl chains of BA impart hydrophobicity to the adhesive, enabling the displacement of the interfacial water layer in underwater environments and allowing direct contact between the adhesive and the substrate. Furthermore, the π–π stacking interactions between benzene rings along the polymer chains effectively enhance the adhesive [48]. In addition, the benzene rings contribute to improved adhesion to wood [49]. P(AA-co-BA-co-PEA)/MMT exhibited superior underwater adhesion strength to P(AA-co-BA-co-PEA) on all six substrates, namely PP, PVC, PTFE, wood, 304 stainless steel, and glass slides (Figure 3a), with values of 135.29 ± 6.45 kPa, 119.73 ± 3.45 kPa, 92.15 ± 5.02 kPa, 120.44 ± 4.80 kPa, 238.00 ± 5.52 kPa, and 66.01 ± 3.01 kPa, respectively. The adhesion strengths on PVC, PTFE, and wood were more than twice those of P(AA-co-BA-co-PEA), while the strengths on PP and glass slides exceeded those of P(AA-co-BA-co-PEA) by 67.48% and 56.27%, respectively. The adhesion strength on 304 stainless steel was comparable to that of P(AA-co-BA-co-PEA). Furthermore, we compared the adhesive performance of P(AA-co-BA-co-PEA)/MMT with that of commercially available adhesives for underwater applications. Sika^®^ Multi Stick exhibits an underwater lap shear strength of 90 kPa on PVC substrates. Additionally, a bio-inspired underwater adhesive developed by researchers at the University of Waterloo—referred to as underwater “superglue”—reports a tensile adhesive strength of up to 80 kPa on different substrates including polymers, ceramics, and metals. In contrast, our P(AA-co-BA-co-PEA)/MMT adhesive achieves good adhesion strengths across all six substrates tested (66.01–238.00 kPa). With respect to both the adhesion strength achieved on individual substrate and the breadth of compatible substrates, our adhesive exhibits performance comparable to, and in some cases superior to, that of the aforementioned commercial products.

Collectively, these results demonstrate that the incorporation of MMT can substantially enhance the adhesive performance without introducing additional adhesion-active functional groups.

Both P(AA-co-BA-co-PEA) and P(AA-co-BA-co-PEA)/MMT exhibited reusable characteristics in the lap-shear tests. After five repeated uses, P(AA-co-BA-co-PEA) retained 73.1%, 79.2%, 61.2%, 77.0%, 62.8%, and 76.1% of its initial adhesion strength on PP, PVC, PTFE, wood, 304 stainless steel, and glass slides, respectively (Figure 3b), indicating good reusability. For P(AA-co-BA-co-PEA)/MMT, the results are shown in Figure 3c. The highest adhesion strength was observed on 304 stainless steel (238.00 kPa), and the repeated-use curve on stainless steel followed a similar trend to that of P(AA-co-BA-co-PEA), with a substantial drop in adhesion strength after the first two adhesion cycles. This is mainly because after cohesive failure, the re-bonded adhesive re-contacts a large amount of water, and the carboxyl groups form hydrogen bonds with water molecules, thereby competing for and reducing the coordination sites with the metal substrate. Nevertheless, even at the fifth adhesion cycle, the strength remained 147.95 kPa, which still holds practical value. During the experiments, cohesive failure occurred on all substrates in the initial adhesion tests, which may be attributed to the fact that MMT also simultaneously enhances the interfacial adhesion [50]. This is likewise beneficial for the overall improvement of adhesion strength. Upon repeated adhesion on PP and PTFE, adhesive failure was observed, with only one fracture surface covered with adhesive residue while the other remained relatively clean. This is because PP and PTFE are low-surface-energy plastics, resulting in lower interfacial adhesion compared to other substrates. As a consequence, adhesive failure occurred first on PP and PTFE, and once adhesive failure took place, the adhesion strength in subsequent lap-shear tests decreased significantly. In contrast, P(AA-co-BA-co-PEA) consistently exhibited cohesive failure throughout the experiments, further confirming that the incorporation of MMT effectively enhances the adhesive and thus improves the adhesion strength.

The 180° peel tests were conducted on P(AA-co-BA-co-PEA) and P(AA-co-BA-co-PEA)/MMT, and the results are presented in Figure 3d. The peel strength of P(AA-co-BA-co-PEA)/MMT reached 107.76 ± 5.12 N/m, which was 23.9% higher than that of the unmodified P(AA-co-BA-co-PEA) (86.97 ± 4.29 N/m). This increase in adhesion strength is also attributed to the enhancing effect of MMT.

### 3.3. Swelling Ratio

**Figure 4:**
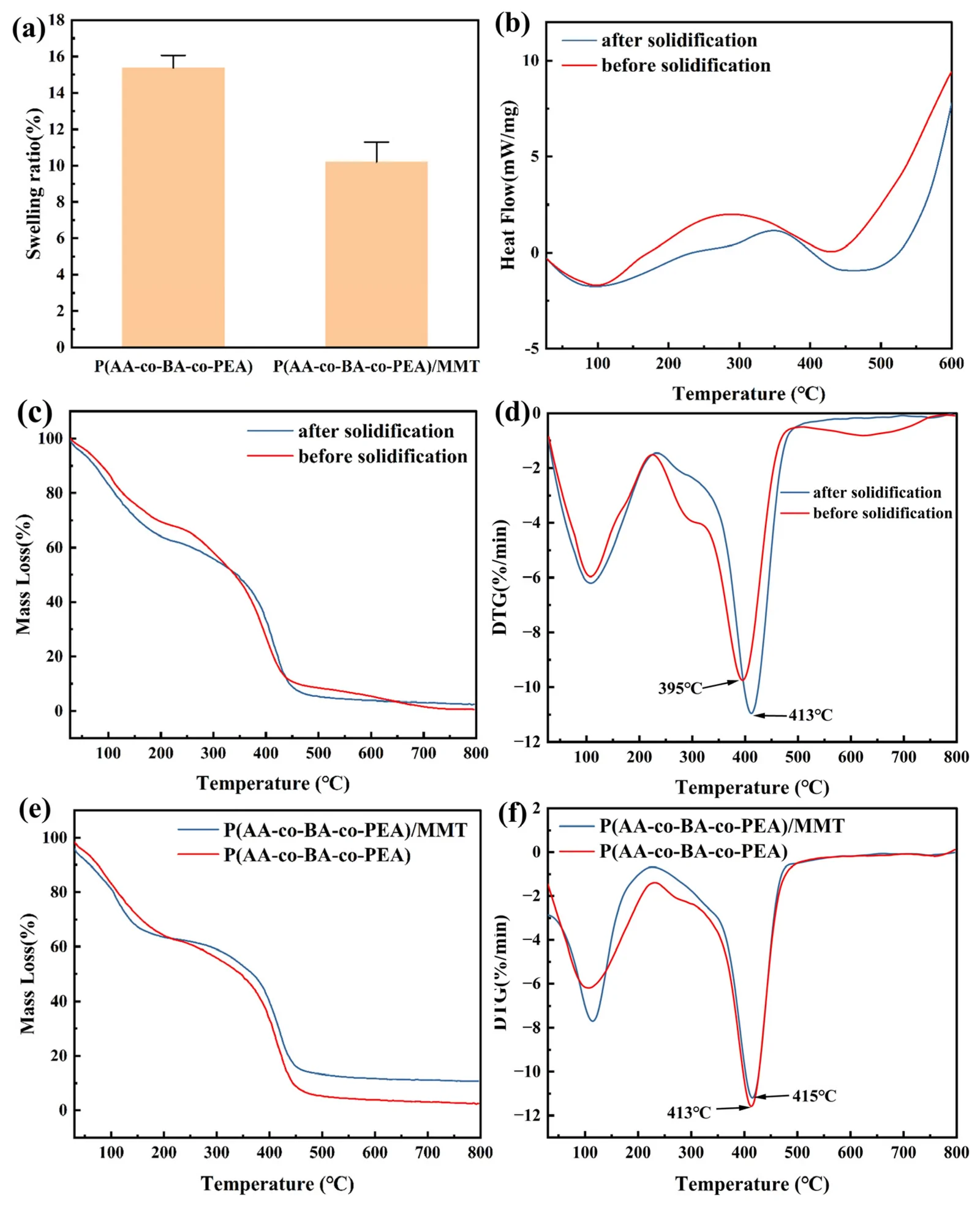
Swelling ratio and thermal performance: (**a**) swelling ratio; (**b**) DSC of P(AA-co-BA-co-PEA); (**c**) TG of P(AA-co-BA-co-PEA); (**d**) DTG of P(AA-co-BA-co-PEA); (**e**) TG of P(AA-co-BA-co-PEA) and P(AA-co-BA-co-PEA)/MMT; (**f**) DTG of P(AA-co-BA-co-PEA) and P(AA-co-BA-co-PEA)/MMT.

Adhesives are prone to swelling in aqueous environments, and excessive water uptake can disrupt the interfacial layer, thereby compromising adhesive–substrate interactions [33]. To evaluate the effect of MMT incorporation on water resistance, the swelling ratios of P(AA-co-BA-co-PEA) and P(AA-co-BA-co-PEA)/MMT were measured, and the results are presented in Figure 4a. The swelling ratio of P(AA-co-BA-co-PEA)/MMT was determined to be 10.20 ± 1.09%, representing a 34.74% reduction compared to that of the unmodified adhesive (15.63 ± 0.70%). This reduction is attributed to the confinement of polymer chain segments within the nanoscale interlayer structure of MMT, which restricts chain segmental mobility, reinforces the physically crosslinked network structure of the adhesive, and suppresses swelling, thereby preserving the integrity of the adhesive–substrate interface and contributing to the improvement of underwater adhesion strength.

### 3.4. Thermal Properties

The improved thermal stability and the enhanced adhesion strength are both associated with the strengthening of intermolecular interactions within the physically crosslinked network structure of the adhesive. Higher thermal degradation temperatures indicate that more energy is required to disrupt these interactions, while the same interactions also contribute to the adhesive’s resistance to failure [51,52]. Accordingly, the P(AA-co-BA-co-PEA) adhesive was characterized by simultaneous thermal analysis before and after solidification, and the results are shown in Figure 4. Comparing the DSC curves before and after solidification (Figure 4b), it was observed that in the low-temperature initial stage, the heat flow values of both samples were negative. However, the heat flow of the adhesive before solidification turned positive at 171 °C, which was significantly earlier than that of the solidified adhesive (234 °C), indicating that the adhesive before solidification began to decompose at a lower temperature. During the thermal decomposition stage, the heat flow of the solidified adhesive remained consistently lower than that before solidification, suggesting that the adhesive before solidification was more prone to violent decomposition, while the solidified sample exhibited greater stability. Throughout the entire testing range, the solidified adhesive demonstrated higher decomposition temperatures and lower total heat release, indicating superior thermal stability.

From the TG curves of P(AA-co-BA-co-PEA) before and after solidification (Figure 4c), it can be observed that weight loss began immediately upon heating to 100 °C. This initial weight loss is primarily attributed to the evaporation of residual DMF and adsorbed moisture, and does not represent the onset of polymer decomposition, which occurs at significantly higher temperatures [53]. The adhesive after solidification exhibited a longer plateau region, confirming the improved thermal stability upon solidification. Comparison of the DTG curves before and after solidification (Figure 4d) revealed that the maximum weight-loss rate temperature of the solidified adhesive was 413 °C, which was higher than that before solidification (395 °C). This observation further corroborates the enhanced thermal stability upon solidification. Collectively, the DSC, TG, and DTG results demonstrate that solidification induces the formation of a physically crosslinked network within the adhesive, which enhances thermal stability and contributes to improved adhesion strength. This is consistent with the fact that the liquid adhesive acquires its adhesion strength only after underwater solidification.

The thermal properties of the P(AA-co-BA-co-PEA)/MMT after solidification are also shown in Figure 4. Comparing the unmodified P(AA-co-BA-co-PEA) with the MMT-modified P(AA-co-BA-co-PEA)/MMT, it can be observed from the TG curves (Figure 4e) that P(AA-co-BA-co-PEA)/MMT exhibited a longer and smoother plateau region, and the residual weight after the plateau was consistently higher than that of P(AA-co-BA-co-PEA), demonstrating that the incorporation of MMT effectively enhanced the thermal resistance of the adhesive. From the DTG curves (Figure 4f), it can be seen that the maximum weight-loss rate temperature of P(AA-co-BA-co-PEA)/MMT (415 °C) was higher than that of P(AA-co-BA-co-PEA) (413 °C). In the temperature range of 150–400 °C, the weight-loss rate of P(AA-co-BA-co-PEA) was consistently higher than that of P(AA-co-BA-co-PEA)/MMT, further demonstrating that the addition of MMT improved the thermal stability of the adhesive. However, the improved thermal stability of the MMT-reinforced adhesive may be attributed to two distinct but coexisting effects: the physical barrier effect of the MMT nanosheets, and the enhanced intermolecular interactions within the polymer network resulting from the physical crosslinking effect of MMT, which require more thermal energy to overcome. The former represents a thermal stabilization mechanism that does not directly reflect adhesion strength, while the latter is closely related to the enhancement of adhesion strength. Combined with the improved adhesion strength observed in the lap-shear tests, it can be inferred that both effects act together, leading to enhanced thermal stability and improved adhesion performance simultaneously [54]. In addition, the final residue of P(AA-co-BA-co-PEA)/MMT was significantly higher than that of P(AA-co-BA-co-PEA). The residual weight of P(AA-co-BA-co-PEA) at 800 °C was 2.49 wt%, while that of P(AA-co-BA-co-PEA)/MMT was 10.75 wt%, from which the MMT content was calculated to be approximately 8.26 wt%, further confirming the incorporation of MMT.

Collectively, the experimental results demonstrate that solidification induces the formation of a physically crosslinked network through hydrophobic aggregation and chain entanglement, leading to significantly improved thermal stability. The incorporation of MMT further reinforces the structural stability of the polymer network, as evidenced by the higher decomposition temperature and extended plateau region. This structural stability provides a structural basis for the improvement of underwater adhesion performance.

### 3.5. Rheological Properties

**Figure 5:**
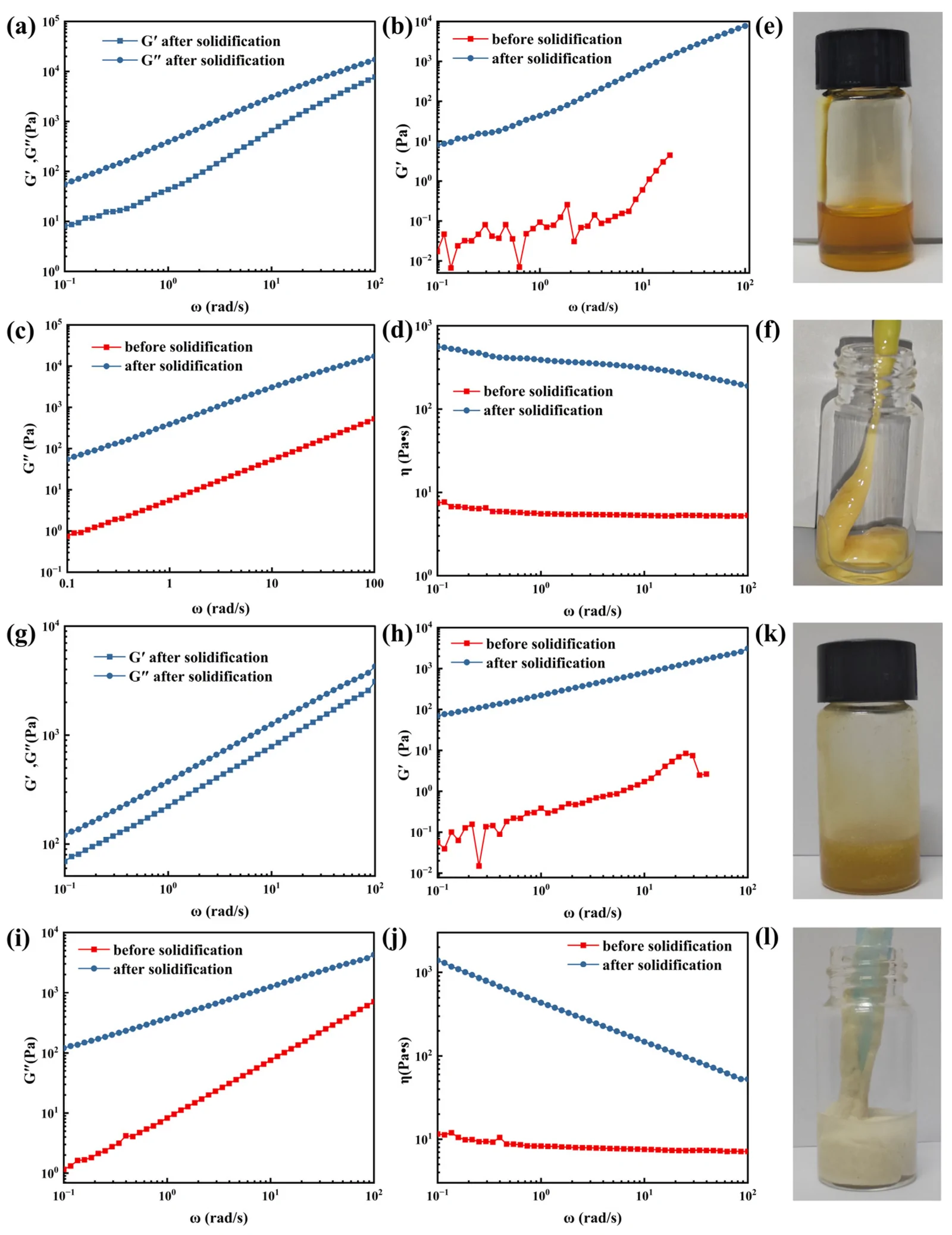
Rheological properties of the adhesives before and after solidification: (**a**) frequency sweep moduli curves of P(AA-co-BA-co-PEA) after solidification; storage modulus (G′) (**b**), loss modulus (G″) (**c**) and complex viscosity (η) (**d**) of P(AA-co-BA-co-PEA) before and after solidification, respectively; photograph of P(AA-co-BA-co-PEA) before (**e**) and after (**f**) solidification, respectively; (**g**) frequency sweep moduli curves of P(AA-co-BA-co-PEA)/MMT after solidification; (**h**) storage modulus (G′) of P(AA-co-BA-co-PEA)/MMT before and after solidification; (**i**) loss modulus (G″) of P(AA-co-BA-co-PEA)/MMT before and after solidification; (**j**) complex viscosity (η) of P(AA-co-BA-co-PEA)/MMT before and after solidification; photograph of P(AA-co-BA-co-PEA)/MMT before (**k**) and after (**l**) solidification, respectively.

Rheological tests were conducted on P(AA-co-BA-co-PEA) and P(AA-co-BA-co-PEA)/MMT before and after solidification for 5 min, and the results are shown in Figure 5. For both adhesives, the storage modulus (G′) and loss modulus (G″) after solidification increased with increasing shear rate, with G″ consistently exceeding G′ (Figure 5a,g), indicating that the adhesives remained in a viscosity-dominated fluid state after brief solidification. However, the absolute values of both G′ and G″ increased substantially. This increase indicates that the intermolecular interactions between polymer chains have been strengthened, chain mobility has been restricted, and a physically crosslinked network structure has begun to form, suggesting an enhanced resistance to deformation of the adhesive [55]. With prolonged solidification time, as solvent exchange proceeds further and hydrophobic aggregation and chain entanglement continue to develop, the absolute values of G′ and G″ are expected to increase further, and the system will gradually transition toward an elasticity-dominated state.

As shown in Figure 5b–d,h–j, the storage modulus (G′), loss modulus (G″), and complex viscosity (η) of both P(AA-co-BA-co-PEA) and P(AA-co-BA-co-PEA)/MMT after solidification were substantially higher than those before solidification. The significant elevation of G′ indicates that solvent-exchange-induced hydrophobic aggregation drove the transition toward a solid-like state, thereby enhancing the elastic resistance of the system to deformation. The substantial rise in G″ reflects a marked increase in intermolecular friction and chain segmental restriction, implying that the solidified network possesses a greater capacity for energy dissipation during flow. The sharp increase in η further confirms that the adhesive transitioned from a free-flowing polymer solution to a high-viscoelastic body with a percolating network structure, which ensures structural integrity in aqueous environments and provides robust support for underwater adhesion. These rheological changes collectively demonstrate the formation of a robust polymer network, which provides essential structural support for reliable underwater adhesion. Moreover, the relatively low modulus and complex viscosity of the adhesive before solidification facilitate spreading and wetting on the substrate surface, allowing adaptation to various surface topographies and maximizing contact area, which is beneficial for underwater adhesion.

Further examination of Figure 5b,h reveals that the storage modulus (G′) of both adhesives before solidification exhibited noticeable fluctuations. This is attributed to the fact that the adhesive is a polymer solution with a broad molecular weight distribution (PDI ≈ 2.9), as confirmed by GPC analysis. Different molecular weight chains contribute differently to the storage modulus: regions with higher molecular weight chains may provide higher G′, while those with lower molecular weight yield lower G′. As frequency increases, chain segments with distinct molecular weights successively respond to the oscillatory shear, leading to stepwise variations in the elastic contribution. Additionally, at intermediate frequencies, the dynamic competition between chain entanglement and disentanglement further complicates the viscoelastic response, resulting in the observed irregularities. Notably, upon incorporation of MMT, the fluctuations in G′ were significantly suppressed and the stability was greatly improved. This behavior suggests that MMT nanosheets served as physical crosslinking points, connecting polymer chains of different molecular weights into a unified network. The collective response of the network replaced the individual responses of isolated chains, thereby achieving a more uniform viscoelastic response across the broad molecular weight distribution. Furthermore, at low shear rates, the G′, G″, and η of P(AA-co-BA-co-PEA)/MMT were all higher than those of P(AA-co-BA-co-PEA). These results collectively confirm that the incorporation of MMT enables the adhesive to integrate polymer chains of different molecular weights into a more uniform network through physical crosslinking even before solidification, thereby laying a foundation for the subsequent improvement of underwater adhesion performance.

The visual observation of the adhesives before and after solidification (Figure 5e,f,k,l) showed that P(AA-co-BA-co-PEA) was a yellow transparent viscous liquid before solidification and became a more viscous yellow opaque fluid after solidification. P(AA-co-BA-co-PEA)/MMT was a yellow turbid liquid before solidification and turned whitish with increased viscosity after solidification. These visual changes correspond to hydrophobic aggregation during the solvent-exchange process, and the apparent increase in viscosity can be directly observed, reflecting the formation of a polymer network structure upon solidification.

### 3.6. Analysis of the Adhesion Mechanism

The liquid adhesive prepared via the one-pot method exhibited sufficient fluidity to spread over the substrate surface and establish intimate contact. Upon contact with water, the adhesive maintained its integrity without dispersing, indicating adequate structural integrity in the as-synthesized state.

**Figure 6:**
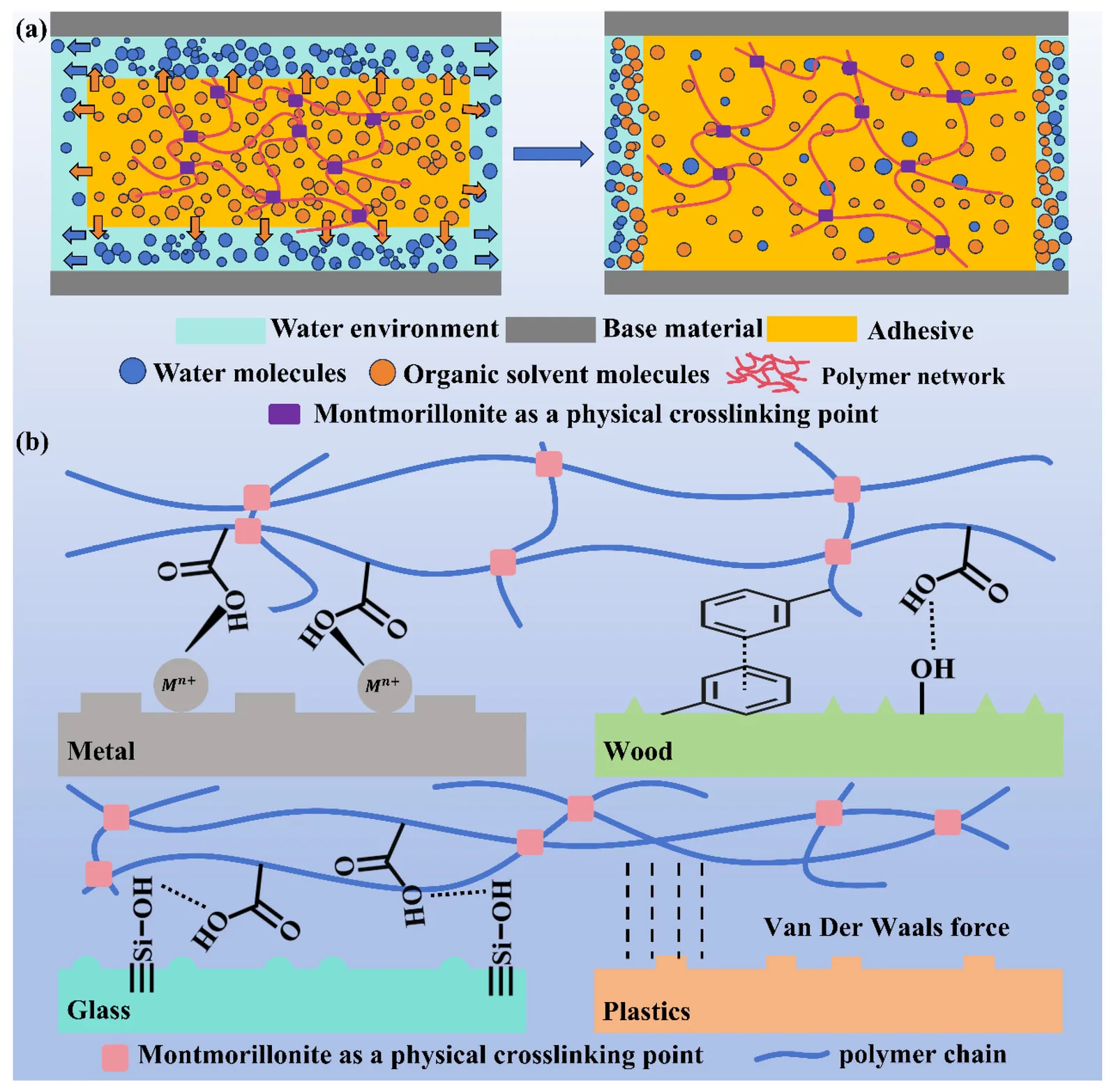
Schematic illustration of the adhesion mechanism of P(AA-co-BA-co-PEA)/MMT: (**a**) solvent exchange displacing the hydration layer; (**b**) interfacial adhesion.

Based on the experimental observations, the following adhesion mechanism is proposed. When the polymer solution is transferred from a good solvent (DMF) to a poor solvent (water), the concentration gradient drives the diffusion of DMF molecules into the aqueous phase. During this process, the hydration layer at the adhesive–substrate interface is displaced, as evidenced by the successful adhesion achieved on various substrates under water. This process is suggested to be facilitated by the accelerating effect of hydrophobic groups on hydration-layer displacement and the promoting effect of hydrophilic carboxyl groups on substrate wetting, thereby enabling intimate contact between the adhesive and the substrate (Figure 6a). As solvent exchange proceeds, the adhesive concentration increases, triggering hydrophobic aggregation [56], which induces phase separation and conformational changes of the polymer, driving the transition toward a solid-like state with enhanced intermolecular interactions (Figure 6a). This interpretation is supported by the observed increase in rheological moduli and the visual change from a transparent liquid to an opaque gel upon solidification.

Subsequently, interfacial adhesion is established through multiple non-covalent interactions, including metal coordination, π–π stacking, hydrogen bonding, and van der Waals forces, depending on the nature of the substrate (Figure 6b). These interactions are well documented in the literature and are considered to account for the broad substrate applicability observed in our adhesion tests.

Furthermore, XRD analysis confirms the formation of an intercalated nanocomposite structure upon MMT incorporation. The intercalated MMT layers are suggested to serve as the added physical crosslinking points that restrict chain mobility and enhance intermolecular interactions. This structural reinforcement is associated with improved underwater adhesion performance in the lap-shear tests.

## 4. Conclusions

In this work, sodium montmorillonite was incorporated into a ternary acrylic copolymer-based underwater adhesive through nanoscale intercalation. The confinement of polymer chains within the MMT interlayers effectively improved the bulk properties of the pristine P(AA-co-BA-co-PEA) adhesive. FTIR, ^1^H NMR, XRD, and GPC collectively confirmed the successful preparation of the P(AA-co-BA-co-PEA)/MMT nanocomposite adhesive. Lap-shear tests demonstrated that the P(AA-co-BA-co-PEA)/MMT adhesive achieved adhesion strengths of 135.29 ± 6.45, 119.73 ± 3.45, 92.15 ± 5.02, 120.44 ± 4.80, 238.00 ± 5.52, and 66.01 ± 3.01 kPa on PP, PVC, PTFE, wood, 304 stainless steel, and glass substrates, respectively, all of which were higher than those of the unmodified P(AA-co-BA-co-PEA) adhesive. Furthermore, the nanocomposite adhesive exhibited good reusability on PVC, wood, and glass substrates. Its peel strength on PTFE was also significantly improved, reaching 107.76 ± 5.12 N m^−1^. Swelling measurements, thermal analysis, and rheological characterization collectively demonstrated that the incorporation of MMT reinforced the physically crosslinked network structure and improved the bulk properties of the adhesive, thereby improving its overall adhesion performance. These findings indicate that MMT intercalation is an effective strategy for reinforcing acrylic copolymer-based underwater adhesives. The resulting P(AA-co-BA-co-PEA)/MMT nanocomposite adhesive exhibits strong adhesion to various substrates, favorable reusability, and considerable potential for practical underwater bonding applications.

## Data Availability

The raw data supporting the conclusions of this article will be made available by the authors on request.

## Supplementary Materials

The following supporting information can be downloaded at: https://www.mdpi.com/article/10.3390/polym18172129/s1, Figure S1. Adhesion strength of P(AA-co-BA-co-PEA)/MMT adhesives prepared with different MMT content loadings on PVC, wood, and 304 steel substrates after solidification for 12 h at room temperature.
