# Supplementary material for: Montmorillonite-Reinforced Acrylic Copolymer Adhesive for Robust Underwater Bonding via Bulk-Interfacial Adhesion Synergy
Source: Polymers (Basel). 2026 Aug 31;18(17):2129. doi: 10.3390/polym18172129 (PMC13568140; doi:10.3390/polym18172129)
Supplement: Supplementary file 1 [file polymers-18-02129-s001.zip › polymers-4468313-supplementary.pdf]

Supplementary Materials

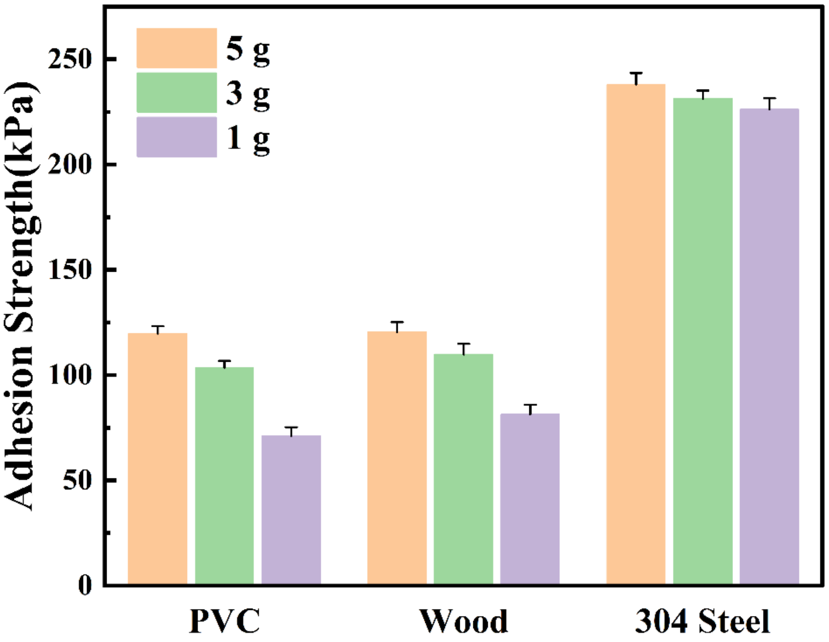

Figure S1. Adhesion strength of P(AA-co-BA-co-PEA)/MMT adhesives prepared with different MMT content loadings on PVC, wood, and 304 steel substrates after solidification for 12 h at room temperature.
